# Selected ethno-medicinal plants from Kenya with *in vitro* activity against major African livestock pathogens belonging to the “*Mycoplasma mycoides* cluster”

**DOI:** 10.1016/j.jep.2016.09.034

**Published:** 2016-11-04

**Authors:** Francisca Kama-Kama, Jacob Midiwo, Joseph Nganga, Naomi Maina, Elise Schiek, Leonidah Kerubo Omosa, George Osanjo, Jan Naessens

**Affiliations:** a Department of Biochemistry, Jomo Kenyatta University of Agriculture and Technology, P.O. Box 62000, Nairobi, Kenya; b Biosciences Eastern & Central Africa – International Livestock Research Institute, Old Naivasha Road, PO Box 30709, 00100 Nairobi, Kenya; c Department of Chemistry, School of Physical Sciences, University of Nairobi, P.O Box 30197, Nairobi, Kenya; d Department of Pharmacology and Pharmacognosy, School of Pharmacy, University of Nairobi, P.O Box 30197, Nairobi, Kenya

**Keywords:** *Mycoplasma mycoides*, Ethnobotany, Antimicrobial activity, Livestock, Ethno-medicinal from plants from Kenya

## Abstract

**Ethnopharmocological relevance:**

Members of ‘*Mycoplasma mycoides* cluster’ are important ruminant pathogens in Africa. Diseases caused by these *Mycoplasma* negatively affect the agricultural sector especially in developing countries through losses in livestock productivity, mortality and international trade restrictions. There is therefore urgent need to develop antimicrobials from alternative sources such as medicinal plants to curb these diseases. In Kenya, smallholder farmers belonging to the Maasai, Kuria and Luo rely on traditional Kenyan herbals to treat respiratory symptoms in ruminants. In the current study extracts from some of these plants were tested against the growth of members of *Mycoplasma mycoides* cluster.

**Aim:**

This study aimed at identifying plants that exhibit antimycoplasmal activities using an ethnobotanical approach.

**Materials and methods:**

Kenyan farmers of Maasai, Luo and Kuria ethnic groups were interviewed for plant remedies given to livestock with respiratory syndromes. The plant materials were thereafter collected and crude extracts prepared using a mixture of 50% of methanol (MeOH) in dichloromethane (CH_2_Cl_2_), neat methanol (MeOH), ethanol (EtOH) and water to yield four crude extracts per plant part. The extracts were tested *in vitro* against five strains of *Mycoplasma mycoides* subsp. *capri*, five strains of *Mycoplasma mycoides* subsp. *mycoides* and one strain of *Mycoplasma capricolum* subsp *capricolum* using broth micro-dilution assays with an initial concentration of 1 mg/ml. Minimum inhibitory concentration (MIC) of the most active extracts were determined by serial dilution.

**Results:**

Extracts from five plants namely: *Solanum aculeastrum*, *Albizia coriaria*, *Ekebergia capensis*, *Piliostigma thonningii* and *Euclea divinorum* exhibited the highest activities against the *Mycoplasma* strains tested. *Mycoplasma mycoides* subsp. *mycoides* were more susceptible to these extracts than *Mycoplasma mycoides* subsp. *capri* and *Mycoplasma capricolum* susp. *capricolum.* The activities of the crude extracts varied with the solvent used for extraction. The MICs mean values of the active extracts varied from 0.02 to 0.6 mg/ml.

**Conclusions:**

The results suggested that these plants could potentially contain antimicrobial compounds that might be useful for the treatment of respiratory diseases in ruminants. Future work should focus on the isolation and identification of the active compounds from the plant extracts that showed interesting activities and evaluation of their antimicrobial and cytotoxic potential.

## Highlights

• Ethno-medicinal Plants from Kenyan Flora for treating ruminants respiratory symptoms.
• *Solanumaculeastrum* and *Albiziacoriaria*with highest antimycoplasmal activities.
• The MICs mean values of the active extracts varied from 0.02–0.6 mg/ml.

## Graphical abstract

**Figure:**
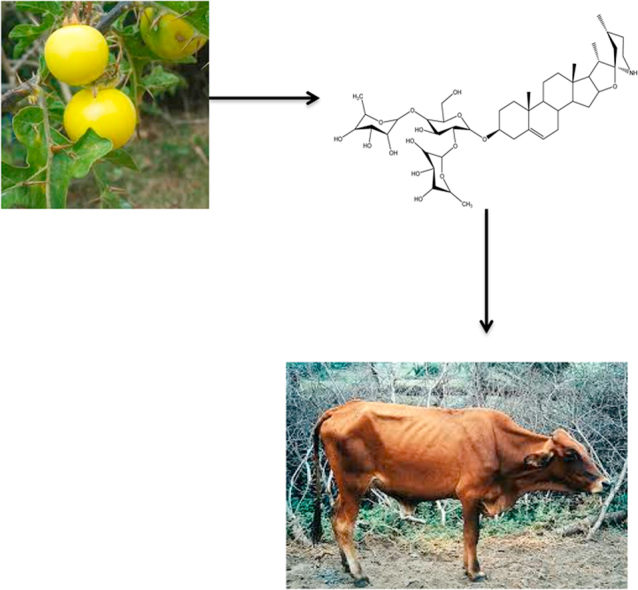

## 1 Introduction

Bacteria of the genus *Mycoplasma* are cell wall-less bacteria and are severe pathogens to humans and animals worldwide. The so-called *‘Mycoplasma mycoides* cluster’ comprises five ruminant pathogens including: *Mycoplasma mycoides* subsp. *mycoides*, *Mycoplasma mycoides* subsp. *capri*, *Mycoplasma capricolum* subsp. *capripneumoniae*, *Mycoplasma capricolum* subsp. *capricolum* and *Mycoplasma leachii* (Fischer et al., 2012). *Mycoplasma mycoides* subsp. *mycoides* and *M. capricolum* subsp. *capricolum* are of highest economic importance as they are known to cause severe respiratory diseases namely: contagious bovine pleuropneumonia (CBPP) and contagious caprine pleuropneumonia (CCPP), respectively. These diseases substantially affect the agricultural sector especially in developing countries through losses in livestock productivity, mortality and international trade restrictions. They are trans-boundary diseases and constitute a threat to disease-free countries (Tambi et al., 2006). Better measures are needed for the progressive control of diseases like CBPP in sub-Saharan Africa (Jores et al., 2013).

The *Mycoplasma* are resistant to many antimicrobials as they lack a cell wall. Antimicrobials widely used to treat infections with *Mycoplasma* include tetracyclines, macrolide-lincosamide-streptogramin-ketolide antibiotic group and fluoroquinolones (Gruson et al., 2005; Renaudin, 2005). Lately, very high resistance levels against macrolides have been reported for the human pathogen *Mycoplasma pneumonia* in China (Cao et al.., 2010). This demonstrates the build-up of acquired resistance within the genus *Mycoplasma* within a relatively short time (Morozumi et al., 2008, Xin et al., 2009). Increasing resistance against antimicrobials calls for the development of new antimicrobial substances. An approach for the development of novel antimicrobials is the use of ethno-botanical information in order to characterize plant substances for their antimicrobial activity. Traditional medicine based on medicinal plants in Africa is not only used in the primary health care system for people living mainly in rural areas in developing countries (Njoroge et al., 2010, Owuor et al., 2012), but also to manage a wide variety of livestock diseases (Githiori et al., 2005; Ole-Miaron, 2003).

In Kenya, farmers such as those belonging to the Maasai, Luo and Kuria ethnic groups already rely on indigenous plants to treat livestock diseases. This is in partly because antimicrobials are costly and not stocked or accessible in many regions of sub-Saharan Africa (Planta, 2007, Tsigemelak et al., 2016). Selected medicinal plants listed by these smallholder farmers belong to different families namely: *Mimosaceae, Canellaceae, Oleaceae, Apocynaceae, Asteraceae, Ebenaceae, Fabaceae, Anacardiaceae, Rhamnaceae, Verbenaceae, Solanaceae, Clusiaceae, Curcubitaceae, Lamiaceae, and Rutaceae.* This study sought to identify plants that exhibit antimycoplasmal activities using an ethno-botanical approach.

## 2 Materials and methods

### 2.1 Identification of medicinal plants used to treat respiratory livestock diseases

An ethnobotanical approach (Cox PA1, 1994; Martin., 2000) was used, whereby farmers were interviewed on their ways of controlling respiratory diseases such as CBPP using medicinal plants. The questionnaire (Supplementary 1) and consent document (Supplementary 2) was translated into the local language of the farmers to be interviewed. Ethical clearance covering a period of one year (January 2014–January 2015) was obtained from the Ethical Review Committee at the Kenya Medical Research Institute, which is registered in Kenya (KEMRI/RES/7/3/1).

**Fig. 1:**
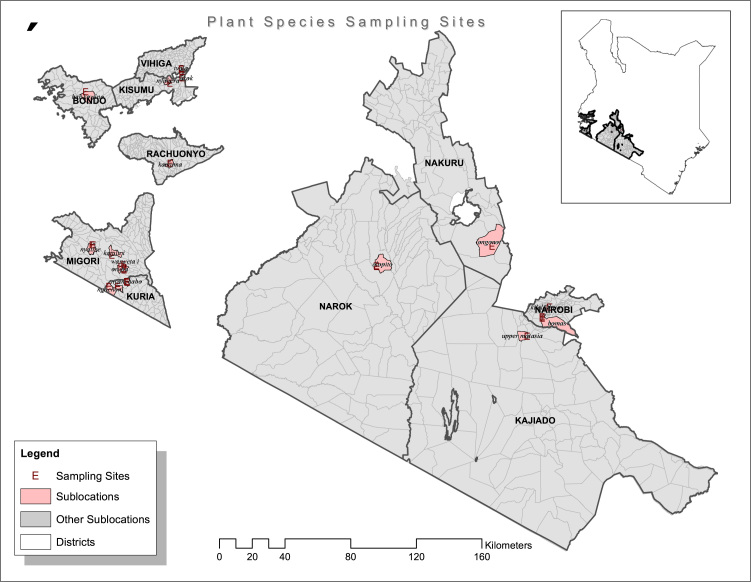
Sample site location map of Kenya.

Livestock farmers belonging to the three ethnic groups namely Massai, Luo and Kuria were interviewed in Nairobi, Kiserian, Narok, Kisumu, Migori and Kuria using a questionnaire. A total of twenty plants (Table 1) were identified and collected from different geographical regions (Fig. 1).

### 2.2 Plant preparation and extraction

Plant parts namely: leaves, roots, stems, stem bark or berries (about 1–2 kg per plant part) were sampled and immediately transported in open polythene bags from the collection site to the Department of Chemistry, School of Physical Sciences (SPS), University of Nairobi. A voucher number for each plant can be found at the School of Biological Sciences (SBS), University of Nairobi Herbarium. Plant parts were dried at room temperature without direct sunlight for three weeks and then ground into fine powder using a MM 20 grinder (Wiley laboratory mill, standard model No. 2, Arthur H. Thomas Company).

### 2.3 Extraction with selected organic solvents

The solvents used for the extraction process included MeOH, ethanol (EtOH) and a mixture of 50% MeOH in CH_2_Cl_2_. The plant material (200 g) was mixed with 500 ml of each of the above solvents in a glass beaker and soaked at room temperature for four hours. After decantation, the insoluble phase was discarded. The supernatant was transferred into a glass flask and concentrated to a volume of about 2 ml using a rotary evaporator (Buchi R114) at 35 °C. The mixture was then transferred into a 10 ml glass tube. The glass flask was rinsed 2–3 times with approximately 1 ml of CH_2_Cl_2_ and the rinse was added to the 2 ml that were harvested before. The concentrated supernatant (about 4 ml) was covered with a perforated aluminium foil and stored to dryness at room temperature.

### 2.4 Extraction with water

Fresh plant material (200 g) was boiled for 15 min and cooled at room temperature. The mixture was transferred into a 50 ml flask and kept in a freezer at −20 °C for 3 days. After three days, the mixture was removed from the freezer and lyophilized using a freeze-drier (Christ Beta 336 Osterode/Harz) for a maximum period of four days. The dried crude extracts were removed from the freeze-dryer and kept aside at room temperature.

### 2.5 Preparation of crude extracts for antimycoplasmal activity tests

Crude extracts (100 mg) from the organic solvents were reconstituted into 1 ml of dimethyl sulfoxide (DMSO) while the same amount of aqueous extracts were reconstituted into 1 ml of water (100%) to make a stock solution of 100 mg/ml. The mixture of each extract was prepared by vortexing to ensure homogenization of the solution.

### 2.6 Bacterial strains and culture conditions

All laboratory manipulations with *Mycoplasma* were carried out under BSL2 conditions (Mwirigi et al., 2016). *Mycoplasma mycoides* subsp. *capri*, *Mycoplasma mycoides* subsp. *mycoides* and *Mycoplasma capricolum* subsp *capricolum* (Table 2, Table 3) were cultured in Pleuropneumonia Like-Organism (PPLO) broth (Difco™ PPLO Broth) media prepared as follows: 21 g of PPLO was dissolved in 700 ml of distilled water and autoclaved for 15 min at 121 °C. The mixture was cooled in a water bath to 55 °C and supplemented with phenol red (Carl Roth GmbH) to a final concentration of 3%, 200 ml horse serum (Sigma), 0.25% of glucose (Carl Roth GmbH), 0.15% of penicillin G (Carl Roth GmbH) and 0.25% of thallium acetate (Carl Roth GmbH). A forty-eight well plate was used to grow *Mycoplasma* strains where they were incubated at 37 °C for a minimum period of seven days. Growth of *Mycoplasma* cells was determined by color change from red to yellow (Stemke and Robertson, 1982), as a result of pH change due to the growth of *Mycoplasma* strains. Stock cultures of both *Mmc*, *Mmm* and *Mcc* liquid cultures were grown to a density of approximately 1–3×10^6^ cells per ml as measured by the colony forming units method (Stemke and Robertson, 1982) and cryopreserved at −80 °C.

### 2.7 Antimicrobial susceptibility testing

The broth microdilution method as described by Arjoon et al. (2012) was used to characterize the *in vitro* antimycoplasmal activity of plant extracts and to determine the minimum inhibitory concentrations employing serial dilutions of the plant extracts. Briefly, a stock solution of 100 mg/ml was prepared and 10 µl (1 mg) of the same was used as the initial concentration mixed with 1 ml of cell culture (1 mg/ml) for the antibacterial test. A control sample consisting of only the solvent included. After seven days of incubation, the cultures were checked for color change from red to yellow, which is an indication of *Mycoplasma* growth resulting from the change of pH. No color change meant that the crude extracts in the well prevented growth of *Mycoplasma*. Data were analysed using Graphprism software version 6.0.

### 2.8 Determination of the minimum inhibitory concentration (MIC)

Active extracts were serially diluted in order to determine the minimum inhibitory concentration (MIC), which was defined as the lowest concentration that inhibited the growth of different *Mycoplasma* strains for an incubation time of seven days to ensure complete inhibition. The MICs of all the active extracts were determined by broth microdilution as previously described by Arjoon et al. (2012) starting from 1 mg/ml to 0.000005 mg/ml. The experiment was repeated thrice for each assay and mean was recorded. The standard error of mean was obtained at P <0.05.

## 3 Results

### 3.1 Interview

The results of the interview with 28 farmers from different parts of Kenya (Fig. 1) showed that most of the farmers rely on medicinal plants to treat ruminant respiratory diseases symptoms such as deep dry cough, extended neck, fever and weight loss. As a result of the interview 20 different plant species used as remedies were identified. The total number of farmers interviewed and that of plants collected are presented in Table 4.

Whilst the interview was based on remedies against livestock respiratory symptoms (Table 5), the farmers’ responses included human diseases treated with these plants (See supplementary table 3).

The current studies show that the most commonly used plants included; *Carissa spinarum, Lantana trifolia and Solanum aculeastrum*. These plants were administered orally in the form of concoctions, mostly in combination with other plants. The infusion of the roots, stem bark and fruits, of one of the active plants, *Carissa spinarum* were administered orally to the livestock, in combination with decoction of other plants such as *Albizia coriaria* (Johns et al., 1990, Owuor et al., 2012). The juice from the berries of *Solanum aculeastrum,* which showed interesting antimicrobial activities, was usually instilled into the cattle, goat or sheep's nostrils (Owuor et al., 2012).

### 3.2 Antimicrobial susceptibility testing

The organic and aqueous extractions from the plant parts yielded a total of one hundred and fifty two extracts (Supplementary 4).

The antimicrobial activity of 152 extracts from the twenty plants (Supplementary 5) selected on the basis of their medicinal uses among the Maasai, Luo and Kuria farmers were evaluated against the growth of five *Mmc*, five *Mmm and one Mcc* strains. The results of the antimycoplasmal activity of the five most active plants are represented in Table 6.

The antimycoplasmal test of twenty ethno-medicinal plants used by these communities showed that only five (5) plants namely: *Solanum aculeastrum*, *Albizia coriaria*, *Ekebergia capensis*, *Piliostigma thonningii* and *Euclea divinorum* inhibited the growth of all the strains tested in the current study. The activities of these extracts differed significantly depending on the plant part and the solvent used for the extraction process. The aqueous extracts from the stem of *Solanum aculeastrum* exhibited the highest antimicrobial activities with MIC values of 0.02 mg/ml at p ≤ 0.05. In general, *Mmm* strains showed that they were more susceptible to the active extracts from five active plants compared to *Mmc* and *Mcc* strains.

Furthermore, it was observed that all organic extracts *ca* 50% MeOH in CH_2_Cl_2_, neat MeOH and EtOH from the berries of *Solanum aculeastrum* inhibited all the *Mmc, Mcc* and *Mmm* strains used while the water extracts from the berries and the stem selectively showed inhibitory effect only on *Mmm* strains.

Similarly, all organic extracts from the stem bark of *Albizia coriaria* showed inhibitory effects against the growth of all *Mycoplasma* strains used in this study while the polar aqueous extracts from the stem bark and leaves inhibited some *Mmm* strains (Table 6).

The extract of the stem bark *Ekebergia capensis* obtained using 50% MeOH in CH_2_Cl_2_ and 100% MeOH were active against the growth of almost all the strains of *Mycoplasma* tested, while those from the stem bark of *Piliostigma thonningii* obtained using MeOH in CH_2_Cl_2_ and 100% MeOH inhibited the growth of all the strains.

It was clear that the EtOH extracts of the active plants except for the stem bark of *Euclea divinorum* and *A. coriaria* and from the berries of *Solanum aculesatrum* were inactive against all *Mmm*, *Mmc,* and *Mcc* strains used in this study. It is likely that the inactivity of the EtOH extracts of most plants was attributed to the nature of the target compounds expected to be polar.

Six plants namely: *Acacia xanthophloea, Warbugia ugandensis, Olea europeae*, *Rhus vulgaris*, *Solanum incanum* and *Tylosema fassoglensis* showed moderate antimycoplasmal activity that differed from one strain to another. The extracts from these plants inhibited almost half of all the *Mmm*, *Mmc* and *Mcc* strains with the exception of extracts from *Tylossema fossoglensis* and *Acacia xenthophloea,* which were active against all *Mmm,* tested in this study.

The plants that had minimal to no activities including; *Gutenbergia cordifolia*, *Carissa spinarum*, *Zizyphus abyssinica, Garcinia buchananii*, *Lantana trifolia*, *Tithonia diversifolia*, *Toddalia asiatica, Fuerstia africana* and *Momordica foetida* were excluded from further studies.

### 3.3 Minimum inhibitory concentration

The minimum inhibitory concentration (MICs) regarded as the lowest concentration of the active extract that inhibited the growth of *Mycoplasma* strains after 7 days of incubation were determined by preparing serial dilutions of the initial concentration. In general, lower MIC mean values were observed with *Mmm* strains compared to *Mmc* and *Mcc* strains. The extracts from the berries and stem bark of *Solanum aculeastrum* obtained using 50% MeOH-CH_2_Cl_2_ and water were the most active with MIC mean values of 0.02 mg/ml. The stem bark extracts from *Albizia coriaria* and *Ekebergia capensis* showed activity with the lowest MIC mean value of around 0.13 mg/ml against *Mmm* strains while that of the extracts of *Euclea divinorum* was active with MIC mean value of 0.5 mg/ml.

The MIC mean values of active extracts against *Mmc* strains ranged from 0.13 mg/ml to 0.60 mg/ml while those against *Mmm* strains ranged from 0.02 to 0.5 mg/ml (Table 7).

## 4 Discussion

It is interesting to note that all the extracts from the two plants frequently mentioned by farmers including: *Carissa spinarum* (10/28) and *Lantana trifolia* (8/28), were not potent against all *Mycoplasma* strains tested. Furthermore, this contradicts, previous reports that have shown that *Lantana trifolia* elaborated flavonoids such as umuhengerin (**1**) with good antimicrobial activity (Rwangabo et al., 1988). The inactivities of the extracts of these two plants could probably be attributed to the fact that traditional medicine practitioners and farmers rarely use these plants singly but rather in combination with others. *Carissa spinarum*, for example, was mostly used in combination with plants like *Albizia coriaria*. Similarly, extracts of *Lantana trifolia* were frequently used in combination with those of *Piliostigma thonningii.*

Most of the plants with interesting *in vitro* antimycoplasmal activities against all strains tested including: *Solanum aculeastrum* (berries), *Albizia coriaria* (stem bark), *Ekebergia capensis* (stem bark), *Piliostigma thonningii* (stem bark) and *Euclea divinorum* (stem bark), have previously been studied for activities against other strains of bacteria such as; *Staphylococcus aureus*, *Escherichia coli*, *S. typhi* (Al-Fatimi et al., 2007, Munyendo et al., 2011, Owuor et al., 2012). However this is the first report of these plants against *Mycoplasma*.

All organic extracts from the berries of *Solanum aculeastrum* were active against all the strains, while the aqueous extracts showed inhibitory activities against only a few strains. Although farmers were not using the stem or leaves of this plant, extracts from these plant parts inhibited selected *Mycoplasma* strains. Previous investigations by Wanyonyi et al. (2003) showed that extracts from *S. aculeastrum* were active against *E. coli* and *S. aureus*. The ethno-botanical survey by Owuor et al. (2006) showed that this plant is traditionally used for the management of both livestock (respiratory symptoms) and human diseases such as gonorrhoea. In a separate report, Koduru et al. (2007) observed that the extracts from the berries and leaves of *S. aculeastrum* were active *against* selected gram-positive and gram-negative bacteria with the concentration values ranging between 4.0 and 10.0 mg/ml, much higher than the initial concentration of 1 mg/ml, in the present study. Furthermore, this plant was also reported to exhibit antifungal activities Koduru et al. (2007). Nevertheless other bioactive alkaloids from this plant could also impact the observed activity in this study since this plant is known to be an alkaloid bearing plant.

The antimicrobial activities observed in this study could be attributed to the presence of compounds previously isolated from the genus *Solanum* such as flavonoid (tiriloside, **2**), alkaloids (solasodine, **3**), steroidal alkaloids (capsimine, **4,** solanudine, **5**), steroidal alkaloids glycosides (solamargine, **6**) (Murakami et al., 1985, Schakirov and Yunusov, 1990, Wanyonyi et al., 2003, Esteves-souza et al., 2003)

**Figure:**
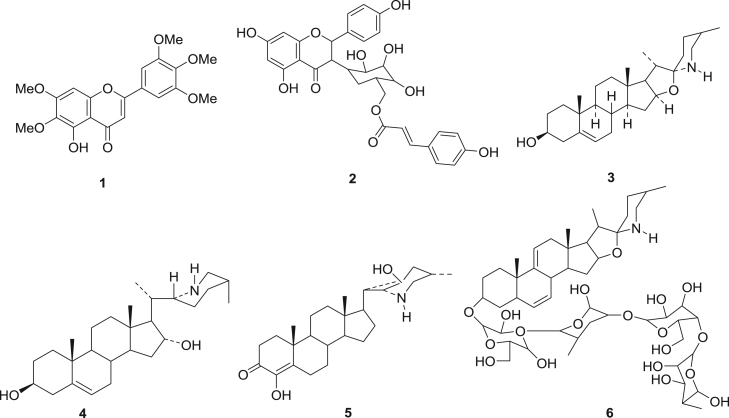

.

The organic extracts from *Albizia coriaria* were active against all the *Mycoplasma* strains used in this study. The presence of compounds such as saponins; albiziatrioside A and B, **7** & **8**, alkaloids; macrocyclic spermine alkaloids, **9** and flavonoids; luteolin, **10**, quercetin-3-O-α-l-rhamnopyranoside, **11** (Mar et al., 1991; Abbel-Kadder et al., 2001; Melek et al., 2011) from the genus *Albizia* could justify the antimycoplasmal activities observed in this study.

**Figure:**
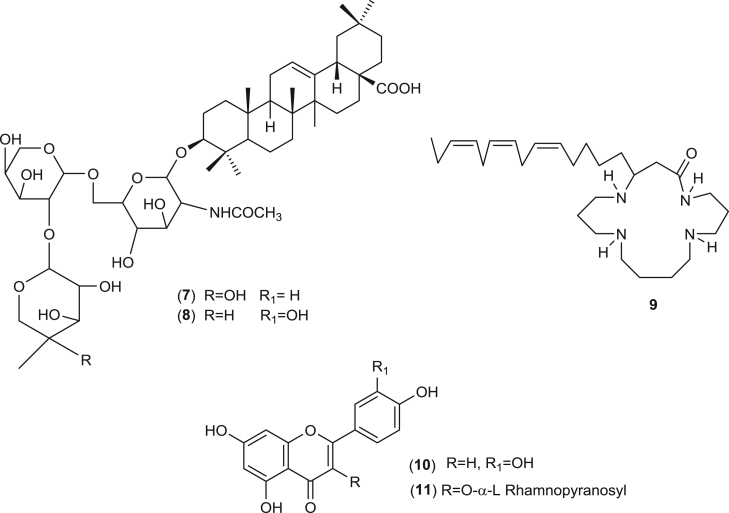

Water extracts from the stem bark of *Albizia coriaria* could not inhibit *Mmc* nor *Mcc* and were active only against few *Mmm* strains. Although farmers do not use the leaves, our study showed that extracts from this plant part inhibited selected *Mmm* strains. Previous studies by Owuor et al., (2012) and Faisal et al. (2012) reported evidence for antimicrobial activity of extracts from the genus *Albizia*, which could possibly also explain the antimycoplasmal activities, observed in the current study. Moreover, secondary metabolites such as: saponins, flavonoids and alkaloids previously reported by Mar et al., (1991); Abbel-Kadder et al., (2001); Melek et al., (2011); Faisal et al., (2012) and Singab et al., (2015) on the genus *Albizia* are known to have antibacterial, antifungal and anticancer activities. The outcome of this study is an indication of the necessity of isolating individual compounds from this plant, which could then be used for the formulation of antimicrobial agents.

All organic extracts from the stem bark and the leaves of *Ekebergia capensis* inhibited the growth of almost all the *Mmm* strains used in this study while *Mmc* and *Mcc* strains were only susceptible to dichloromethane and methanol mixture or methanol extracts from the stem bark of this plant. Little is known on the antimicrobial activities of this plant, but Sewram et al., (2000) reported that compounds isolated from this plant showed uterotonic activities against both pregnant and non pregnant guinea pig uterine smooth muscle. However, compounds previously isolated from the genus *Ekebergia* such as terpenoids; oleanonic acid, **12**, ekeberin A, **13**, flavonoids; kaempferol-3-O-β-d-glucopyranoside, **14**, quercetin-3-O-β-d-glucopyranoside, **15** (Irungu et al., 2014;) could be the reason why these extracts showed good antimycoplasmal activities against all the strains tested

**Figure:**
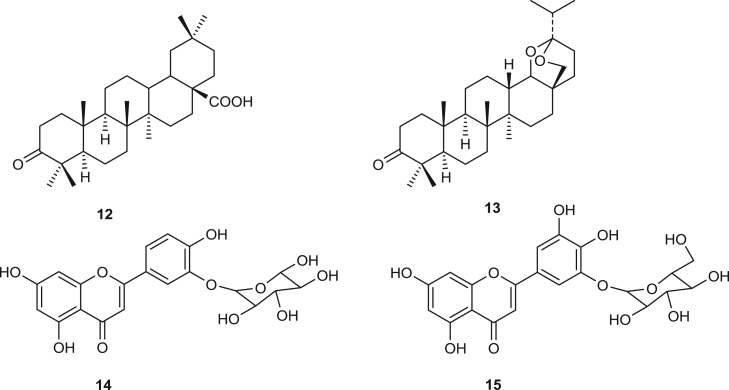

.

The extracts from *Piliostigma thonningii* (50% MeOH in CH_2_Cl_2_ mixture and neat MeOH) inhibited the growth of almost all the strains although not much is known about the antimicrobial properties of this plant. Previous reports on preliminary phytochemical screening of extracts from this plant by Ukwuani et al. (2012) showed that this plant contains compounds such as tannins, saponins, terpenoids, glycosides, flavonoids and alkaloids which could be responsible for the antimycoplasmal activity observed the current study.

For *Euclea divinorum*, only ethanol extracts inhibited the growth of all the *Mmc*, *Mmm* and *Mcc* strains used in this study. This plant has previously been reported to have antimicrobial properties (Orwa et al., 2008), which bolsters our findings and its uses among Kenyan farmers.

Previous phytochemical reports showed that phenolic compounds such as; Eucleanal A and B, **17** &**18** from *Euclea divinorum* (Nishiyama et al., 1996, Ng’ang’a et al., 2012) which could be responsible for the observed antibacterial activities.

The extracts from the rest of the plants showed weak activities against some *Mycoplasma* strains. The low antimycoplasmal activities by these extracts could be due to the fact that the active principles were minor constituents in the extract. Alternatively, the constituent compounds could be acting antagonistically therefore reducing the resultant activities of these extracts.

Surprisingly, a plant such as *Momordica foetida* (whole plant) mentioned by farmers, was inactive against all *Mmc* and *Mmm* strains contrary to previous reports showing that related species such as; *Momordica balsamina* exhibited antimicrobial properties (Otimenyin et al., 2008). This could be due to the unique nature of *Mycoplasma* strains which unlike other bacteria strains lack a cell wall and have a small genome.

From the MIC values observed in this study, it is clear that extracts from the berries and stem bark of *S. aculeastrum* showed good activity suggesting that they could be having bioactive compounds.

## 5 Conclusions and recommendations

From the *in vitro* screening of a total of 152 extracts from 20 medicinal plants selected by Maasai, Kuria and Luo ethnic groups in Kenya, those of five plants, including: *S. aculeastrum* (berries), *A. coriaria* (stem bark), *E. capensis* (stem bark), *P. thonningii* (stem bark) and *E. divinorum* (stem bark) were active against all the *Mmc*, *Mcc* and *Mmm* strains tested in this study. The outcome of the present study should be shared with the smallholder farmer especially those who undertook the interview for effective management of livestock mycoplasmal infections. Further, investigations of the active extracts should be carried out to characterize the phytochemistry that would be responsible for their potencies towards drug development. In addition, the safety profiles of these extracts should be determined before their formulations into phytomedicines against mycoplasmal infections.

## Author contribution

FK-K, GO, JNG, JM, ES and JN conceived and designed the experiments.

FK-K performed the experiments.

FK-K, ES and JN analysed the data.

FK-K, JN and JM contributed reagents/materials/analysis tools.

FK-K, LKO, JM, ES, NM and JN wrote the paper.

## Figures and Tables

**Fig. S1:**
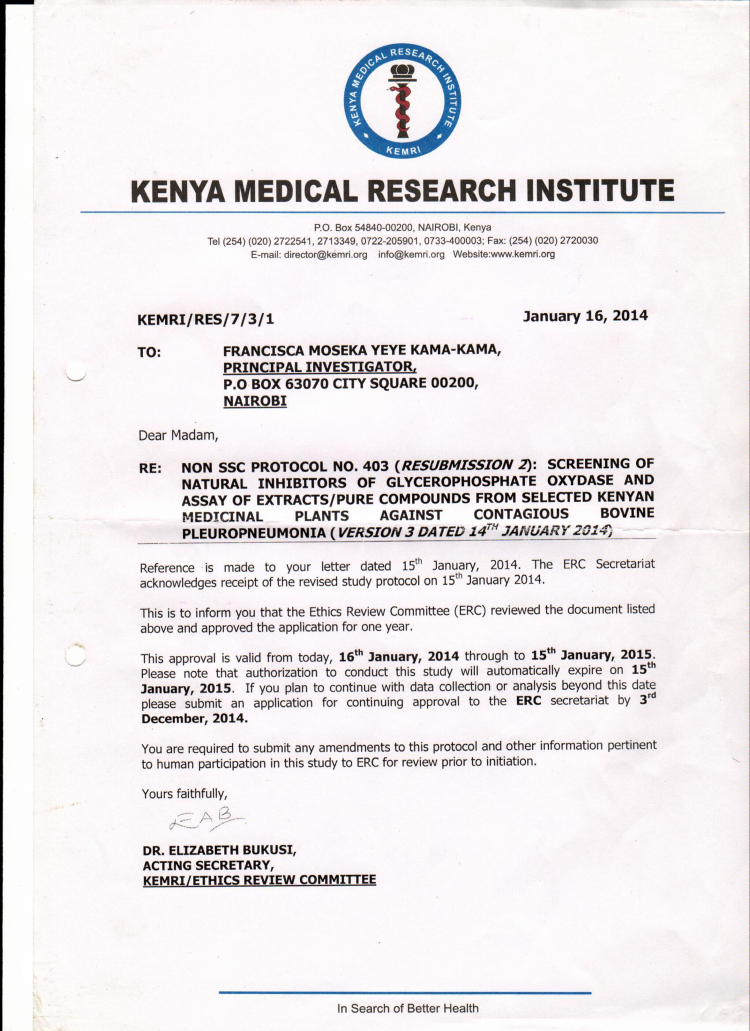
Ethical clearance.

**Table 1:** List of selected medicinal plants collected.

Plant/Family/voucher number	Part used	Ethnic group	Local name
*Acacia xanthophloea/Mimosaceae/Fmk2012/01*	Stem bark	Maasai	Olerai
*Albizia coriaria/Mimosaceae/Fmk2012/02*	Stem bark	Luo, Kuria and Maasai	Ober, Lotoligo, Olerai
*Warbugia ugandensis/Canellaceae/Fmk2012/03*	Stem bark, roots and leaves	Maasai	Ol-sogunoi
*Olea europaea* subsp. *africana/Oleaceae/Fmk2012/04*	Stem and root barks	Maasai	OL-orien
*Ekebergia capensis/Meliaceae/Fmk2012/05*	Stem bark	Luo, Kuria	Tido
*Carissa spinarum/Apocynaceae/Fmk2012/06*	Stem bark, roots and fruits	Luo, Kuria	Ochuoga, Omunyore
*Tithonia diversifolia/Asteraceae/Fmk2012/07*	Stem and leaves	Luo, Kuria	Maua makech, Irita kunguha
*Euclea divinarum/Ebenaceae/Fmk2012/08*	Stem bark	Luo, Kuria	Ochond radoho/Ochol, Ikimusi
*Piliostigma thonningii/Fabaceae/Fmk2012/09*	Stem bark, roots, twigs and leaves	Luo, Maasai, Kuria	Otagalo/Ogalo, Olsagararam, Egekobure
*Rhus vulgaris/Anacardiaceae/Fmk2012/10*	Roots and fruits	Luo, Kuria and Maasai	Sangla maduong, Sangla, Ikinyororio, Engarani
*Ziziphus abyssinica/Rhamnaceae/Fmk2012/11*	Roots, aerial part	Luo, Kuria	Lang’o
*Gutenbergia cordifolia/Asteraceae/Fmk2012/12*	Leaves	Luo, Kuria	Akech, Ikiburua/Ikiburia
*Lantana trifolia/Verbenaceae/Fmk2012/13*	Aerial part and/or the whole plants	Luo, Kuria	Nyabend winyo, Kehembwe/kebarisia
*Solanum aculeastrum/Solanaceae/Fmk2012/14*	Fruits, roots	Maasai, Luo and Kuria	Osigawai, Ochok, Iri botoboto
*Garcinia buchananii/Clusiaceae/Fmk2012/15*	Stem bark, roots	Luo	Onjak
*Tylosema fassoglensis/Fabaceae/Fmk2012/16*	Roots	Luo, Kuria	Ombasa, Omombara
*Mormondica foestida/Cucurbitaceae/Fmk2012/17*	Aerial parts	Luo	Omobora
*Fuerstia Africana/Lamiaceae/Fmk2012/18*	Aerial part	Kuria	Ekebunga baare
*Toddalia asiática/Rutaceae/Fmk2012/19*	Stem bark, roots and leaves	Luo, Kuria	Nyalwet-kwach, Urunisia
*Solanum incanum/Solanacea/Fmk2012/20*	Fruits, roots and leaves	Maasai, Luo and Kuria	Endulelei, Ochok, Iritorotoro

**Table 2:** List of *Mmc* and *Mcc* strains used in this study.

Strains designation	Species	Country of origin	Year of isolation	Host	Data Bank/Strain collection
Y- Goat	*Mmc*	USA	1979	Goat	GenBank
G1313.94	Mmc	Germany	1994	Sheep	MH
G1255-94	*Mmc*	Berlin	1994	Sheep	MH
M-18	*Mmc*	Croatia	1988	Goat	CS
95010-C1	*Mmc*	France	1995	Goat	JF
6443-90	*Mcc*	France	1990	Goat	JF

**Table 3:** List of *Mmm* used in this study.

Strains designation	Species	Country of origin	Year of isolation	Host	Data Bank/Strain collection
PG1	*Mmm*	Africa	1931	Cattle	JF
V5	*Mmm*	Australia	1935	Vaccine strain	JF
B237	*Mmm*	Kenya	1997	Cattle	HW
Afade	*Mmm*	Cameroon	1968	Cattle	JF
Gladysdale	*Mmm*	Australia	1953	Cattle	JF

*References about all these strains can be found in Fischer's paper (Fischer et al., 2012).

**Table 4:** Results of the Interview.

Interviewee	Ethnic group	Collection site	*Acacia xenthophloeae*	*Albizia coriaria*	*Carissa spinarum*	*Ekebergia capensis*	*Euclea divinarum*	*Fuerstia africana*	*Garcinia buchananii*	*Guternbergia cordifolia*	*Lantana trifolia*
I	M	1				X					
II	M	2									
III	M	3		X							X
IV	M	4									
V	M	5									
VI	M	5									
VII	K	6		X				X			X
VIII	K	7	X				X				
IX	K	8			X						
X	K	9								X	
XI	K	10			X					X	X
XII	K	11			X						X
XIII	K	11		X				X			
XIV	K	11							X		
XV	K	11	X		X						
XVI	L	12			X	X	X	X	X	X	
XVII	L	12		X	X			X			X
XVIII	L	13			X			X	X	X	X
XIX	L	14	X		X						X
XX	L	14							X		
XXI	L	14		X	X			X			X
XXII	L	15			X	X	X				
XXIII	L	15									
XXIV	L	15	X								
XXV	L	16	X								
XXVI	L	16	X								
XXVII	L	17									
XXVIII	L	17	X								
No. of plant mentioned			6	5	10	3	3	6	4	4	8


*Mormondica foetida*	*Olea europaea*	*Pilostigma thonningii*	*Rhus vulgaris*	*Solanum aculeastrum*	*Solanum incanum*	*Tithonia diversifolia*	*Toddalia asiatica*	*Tylossema fassoglensis*	*Warbugia ugandensis*	*Ziziphus abyssinica*	No of plants reported
								X			2
		X									1
											2
											0
										X	1
							X				1
											3
				X							3
							X				2
											1
											3
											2
											2
						X					2
						X	X			X	5
				X		X	X	X			10
		X		X		X		X		X	9
						X	X			X	8
		X		X		X	X				7
								X			2
										X	5
				X							4
				X							1
			X							X	2
X											2
X											
	X								X		2
			X	X	X						4
2	1	3	2	7	1	6	6	4	1	5	

I –XVIII: Numbers of respondents; 1-17: Collection sites; X: Collected plant; M: Maasai; K: Kuria; L: Luo

**Table 5:** Information on plants used to treat livestock diseases by the Maasai, Luo and Kuria communities.

Plant/Family/voucher number	Part used	Ethnic group	Local name	Disease treated	Mode of preparation
*Acacia xanthophloea/Mimosaceae/Fmk2012/01*	Stem bark	Maasai	Olerai	Unspecified livestock diseases and coughs	Infusion of the stem bark is administered to the sick animals
*Albizia coriaria/Mimosaceae/Fmk2012/02*	Stem bark	Luo, Kuria and Maasai	Ober, Lotoligo, Olerai	Livestock respiratory diseases	Bark infusion for livestock used as treatment for livestock respiratory diseases
*Warbugia ugandensis/Canellaceae/Fmk2012/03*	Stem bark, roots and leaves	Maasai	Ol-sogunoi	Livestock respiratory diseases symptoms	Infusion of the stem and Roots bark for livestock respiratory symptoms
*Olea europeae/Oleaceae/Fmk2012/04*	Stem and root barks	Maasai	OL-orien	Contagious Bovine Pleuropneumonia	Root and stem bark infusion for CBPP
*Ekebergia capensis/Meliaceae/Fmk2012/05*	Stem bark	Luo, Kuria	Tido	Respiratory symptoms in livestock and remedy for worms	Stem infusion for livestock diseases
*Carissa spinarum/Apocynaceae/Fmk2012/06*	Stem bark, roots and fruits	Luo, Kuria	Ochuoga, Omunyore	Unspecified livestock diseases	An infusion of stem bark is treatment for livestock diseases
*Tithonia diversifolia/Asteraceae/Fmk2012/07*	Stem and leaves	Luo, Kuria	Maua makech, Irita kunguha	Management of weakness in livestock	An infusion of the stems is used as remedy for the management of livestock weakness
*Euclea divinarum/Ebenaceae/Fmk2012/08*	Stem bark	Luo, Kuria	Ochond radoho/Ochol, Ikimusi	Treat join pain in livestock	Decoction of the stem back an pain killer for livestock
*Piliostigma thonningii/Fabaceae/Fmk2012/09*	Stem bark, roots, twigs and leaves	Luo, Maasai, Kuria	Otagalo/Ogalo, Olsagararam, Egekobure	Livestock respiratory symptoms	Infusion of the bark of stem and roots is used as remedy for livestock respiratory symptoms
*Rhus vulgaris/Anacardiaceae/Fmk2012/10*	Roots and fruits	Luo, Kuria and Maasai	Sangla maduong, Sangla, Ikinyororio, Engarani	Livestock weakness	Roots and fruit decoction for give strength to livestock
*Ziziphus abyssinica/Rhamnaceae/Fmk2012/11*	Roots, aerial part	Luo, Kuria	Lang’o	Contagious bovine pleuropneumonia	Decoction of the roots combined with leaves infusion is used to treat CBPP
*Gutenbergia cordifolia/Asteraceae/Fmk2012/12*	Leaves	Luo, Kuria	Akech, Ikiburua/Ikiburia	Livestock unspecified conditions,	Leaves are pounded and administered to livestock, cattle producing hard dung drenched infusion as remedy
*Lantana trifolia/Verbenaceae/Fmk2012/13*	Aerial part and/or the whole plants	Luo, Kuria	Nyabend winyo, Kehembwe/Kebarisia	Treatment for livestock join problems	Leaves infusion is used for livestock joint problems
*Solanum aculeastrum/Solanaceae/Fmk2012/14*	Fruits, roots	Maasai, Luo and Kuria	Osigawai, Ochok, Iri botoboto	Livestock respiratory symptoms and contagious bovine pleuropneumonia	Juice from the fruits is administered in drops to a sick animal
*Garcinia buchananii/Clusiaceae/Fmk2012/15*	Stem bark, roots	Luo	Onjak	Livestock respiratory symptoms	A decoction of the stem bark is used as a remedy for livestock respiratory symptoms
*Tylosema fassoglensis/Fabaceae/Fmk2012/16*	Roots	Luo, Kuria	Ombasa, Omombara	Fever and joint pains in livestock	Root infusion is used to treat livestock
*Mormondica foestida/Curcubitaceae/Fmk2012/17*	Aerial parts	Luo	Omobora	Coughs in livestock	A concoction is considered as remedy for livestock
*Fuerstia Africana/Lamiaceae/Fmk2012/18*	Aerial part	Kuria	Ekebunga baare	Livestock unspecified conditions	Concoction of the aerial part is used as remedy for livestock
*Toddalia asiática/Rutaceae/Fmk2012/19*	Stem bark, roots and leaves	Luo, Kuria	Nyalwet-kwach, Urunisia	Livestock joint pains, coughs, fever	Roots and stem bark concoction is used to treat livestock
*Solanum incanum/Solanacea/Fmk2012/20*	Fruits, roots and leaves	Maasai, Luo and Kuria	Endulelei, Ochok, Iritorotoro	Livestock unspecified conditions	Roots decoction used as remedy for livestock

**Table 6:** Anti mycoplasmal activities of the most active plants.

Plant names	Plant part/ Solvent of extraction	*Mycoplasma mycoides***susp**. *mycoides* (*Mmm*)					*Mycoplasma mycoides***susp**.capri (*Mmc*)					*Mycoplasma capricolum***susp**.*capricolum* (Mcc)
		Afadé	B 237	Gladysdale	PG1	V5	Y-Goat	95010	G1313.94	M-18	G1255/94	6443-90
*E. divinorum*	Stem bark/EtOH	+	+	+	+	+	+	+	+	+	+	+
*P. thonningii*	Stem bark/CH_2_Cl_2_-MeOH (1:1)	+	+	+	+	+	+	+	+	+	+	+
*P. thonningii*	Stem bark/MeOH	+	+	+	+	+	+	+	+	+	+	+
*E. capensis*	Stem bark/ CH_2_Cl_2_-MeOH (1:1)	+	+	+	+	+	−	+	+	+	+	+
*E. capensis*	Stem bark/ MeOH	+	+	+	+	+	+	+	+	+	+	+
*S. aculeastrum*	Leave/ Water	−	+	−	+	+	−	−	−	−	−	−
*S. aculeastrum*	Leave/ EtOH	−	+	+	+	+	−	−	−	−	−	−
*S. aculeastrum*	Leave/MeOH	−	+	−	+	+	−	−	−	−	−	−
*S. aculeastrum*	Leave/CH_2_Cl_2_ -MeOH (1:1)	−	+	+	+	+	−	−	−	−	−	−
*S. aculeastrum*	Berries/Water	+	+	+	+	+	−	−	−	−	−	+
*S. aculeastrum*	Berries/EtOH	+	+	+	+	+	+	+	+	+	+	+
*S. aculeastrum*	Berries/MeOH	+	+	+	+	+	+	+	+	+	+	+
*S. aculeastrum*	Berries/CH_2_Cl_2_-MeOH (1:1)	+	+	+	+	+	+	+	+	+	+	+
*A. coriaria*	Stem bark/Water	−	+	+	+	+	−	−	−	−	−	−
*A. coriaria*	Stem bark/EtOH	+	+	+	+	+	−	−	+	−	−	−
*A. coriaria*	Stem bark/MeOH	+	+	+	+	+	+	+	+	+	+	+
*A. coriaria*	Stem bark/CH_2_Cl_2_-MeOH (1:1)	+	+	+	+	+	+	+	+	+	+	+
*Controls*	Tetracycline	+	+	+	+	+	+	+	+	+	+	+
	Positive control	−	−	−	−	−	−	−	−	−	−	−

Keys: +(Activity); − (No activity); CH_2_Cl_2_ (Dichloromethane); MeOH (Methanol); EtOH (Ethanol).

**Table 7:** Minimal inhibitory concentrations (MICs) of the most active crude extracts.

Plant name	Parts used	Solvent of extraction	Mean of MIC against *Mmm* strains (mg/ml±SEM)	Mean of MIC against *Mmc* strains (mg/ml±SEM)	MIC against *Mcc* strains (mg/ml±SEM)
*A. coriaria*	Stem bark	CH_2_Cl_2_/MeOH (1:1)	0.32±0.110	0.13±0.092	0.05
*A. coriaria*	Stem bark	MeOH	0.13±0.092	0.41±0.090	0.005
*A. coriaria*	Stem bark	EtOH	0.22±0.114	0.23±0.110	0.05
*S. aculeastrum*	Berries	CH_2_Cl_2_/MeOH (1:1)	0.023±0.110	0.23±0.110	0.05
*S. aculeastrum*	Berries	MeOH	0.23±0.011	0.14±0.090	0.005
*S. aculeastrum*	Berries	EtOH	0.13±0.299	0.23±0.110	–
*S. aculeastrum*	Berries	Water	0.22±0.114	0.51±0.150	0.5
*S. aculeastrum*	Stem bark	Water	0.02±0.011	0.60±0.100	–
*P. thonningii*	Stem bark	CH_2_Cl_2_/MeOH (1:1)	0.5±0.000	0.41±0.090	0.05
*P. thonningii*	Stem bark	MeOH	0.5±0.000	0.23±0.110	0.05
*E. capensis*	Stem bark	CH_2_Cl_2_MeOH (1:1)	0.13±0.092	0.40±0.099	0.5
*E. capensis*	Stem bark	MeOH	0.40±0.186	0.23±0.110	0.5
*E. divinorum*	Stem bark	EtOH	0.5±0.000	0.41±0.090	0.5

Controls	Tetracycline	N/A	0.005	0.005	0.005
	Media (PPLO)	–	–	–	–

Keys: CH_2_Cl_2_ (Dichloromethane); MeOH (Methanol); EtOH (Ethanol); P<0.05; SEM (Standard error of mean); N/A (Not Applicable), – (Not active).

## Appendix A Supplementary material

Supplementary material. Questionnaire used for the interview.

.

Supplementary material. Consent form.

.

Supplementary material. Other human conditions treated by the twenty selected medicinal plants.

.

Supplementary material. Results of the total number of the crude extracted from the selected twenty plants.

.

Supplementary material. Results of antimycoplasmal activity tests of all 152 extracts against five strains of Mmc, five strains of Mmm and one Mcc.

.

Supplementary material. **Luo translation**.

.

Supplementary material. Maasai translation.
