# Supplementary material for: Selected ethno-medicinal plants from Kenya with in vitro activity against major African livestock pathogens belonging to the “Mycoplasma mycoides cluster”
Source: J Ethnopharmacol. 2016 Nov 4;192:524–34. doi: 10.1016/j.jep.2016.09.034 (PMC5081062; doi:10.1016/j.jep.2016.09.034)
Supplement: Supplementary file 1 — Supplementary material. Questionnaire used for the interview. [file mmc1.docx]

**PLANT COLLECTION QUESTIONNAIRE**

Participants will take part on a voluntary basis and not be paid. There will be no pressure on them to answer the questions and they can refuse to answer any questions they do not feel comfortable with.

**INTERVIEW GUIDE NUMBER ---------------------------------------------------------------------------------------------------------------------------------------------------------------------------------------------**

**DATE --------------------------------------------------------------------------------------------------------------------------------------------------------------------------------------------------------------------------------**

**LOCATION-------------------------------------------------------------------------------------------------------------------------------------------------------------------------------------------------------------------------**

**PART** **I**

**Section 1: The interviewee**

Name of the interviewee (this is optional) --------------------------------------------------------------------------------- -----------------------------------------------------------------------------------------------------

Locality -------------------------------------------------------------------------------------------------------------------------------------------------------------------------------------------------------------------------------

Age ------------------------------------------------------------------------------------------------------------------------------------------------------------------------------------------------------------------------------------

Sex :                  􀁔 Male  􀁔 Female

Ethnic origin

---------------------------------------------------------------------------------------------------------

Have you attended school? Yes 􀁔 No 􀁔

What is your highest degree?

KCPE 􀁔 KCSE 􀁔 Diploma/certificate of higher education 􀁔 BSC 􀁔

**Section 2: The disease**

Do you have cattle?

Yes 􀁔 No 􀁔

Do you know CBPP?

Yes 􀁔 No 􀁔

Does your cow suffer from the following symptoms? (Fever, deep and dry cough, extended neck, legs apart and weight lost)

Yes 􀁔 No 􀁔

What do you use to treat your cow with in case of the above symptoms?

Medicinal plants 􀁔 Modern antibiotics 􀁔 Both 􀁔 None 􀁔

**PART II**

**Medicinal plants**

What is the name of the medicinal plant that you use?

NB: The identification of plants was done by Mr Mutiso who is a plant taxonomist, school of biological sciences/ University of Nairobi

-----------------------------------------------------------------------------------------------------------

What is/are the name(s) of the pant(s) part(s) used?

-----------------------------------------------------------------------------------------------------------------------------------------------------------------------------------------------------------------------------------------

----------------------------------------------------------------------------------------------------------------------------------------------------------------------------------------------------------------------------------------

How is /are the plant parts prepared?

------------------------------------------------------------------------------------------------------------------------------------------------------------------------------------------------------------------------------------------

How many doses do you administered to your sick cow per day?

-----------------------------------------------------------------------------------------------------------------------------------------------------------------------------------------------------------------------------------------

What are the others medicinal uses of the plants you have mentioned above?

-----------------------------------------------------------------------------------------------------------------------------------------------------------------------------------------------------------------------------------------**Thank you very much for your cooperation**
