# Supplementary material for: Selected ethno-medicinal plants from Kenya with in vitro activity against major African livestock pathogens belonging to the “Mycoplasma mycoides cluster”
Source: J Ethnopharmacol. 2016 Nov 4;192:524–34. doi: 10.1016/j.jep.2016.09.034 (PMC5081062; doi:10.1016/j.jep.2016.09.034)
Supplement: Supplementary file 2 — Supplementary material. Consent form. [file mmc2.docx]

**Title: Traditional Kenyan Health Remedies Used to Treat Respiratory Symptoms in Ruminants Showed In Vitro Activities against members of “*Mycoplasma mycoides cluster*”**

**Investigators**

Francisca Kama-Kama (PhD student at JKUAT and Principal investigator)

Dr Jan Naessens (Co-investigator and supervisor, ILRI)

Dr Joseph Nganga (Co-investigator and supervisor, JKUAT)

**Study location: JKUAT/ILRI**

**Purpose of the research**

The aim of this study is to find alternative therapeutic or prophylactic drugs against contagious bovine pleuropneumonia

**Description of the study**

*Mycoplasma* species belonging to the so-called *‘Mycoplasma mycoides* cluster’ and are all important ruminant pathogens. They are the causal agents of both goats and cattle lung diseases; Contagious Bovine Pleuropneumonia and Contagious Caprine Pleuropneumonia (CBPP and CCPP). CBPP and CCPP are major livestock diseases and impact the agricultural sector specifically in developing countries through livestock product trade, food-supply reduction and decrease productivity. In this project, we will be focusing on contagious bovine pleuropneumonia (CBPP) which is one of the major infectious diseases affecting cattle in Africa. CBPP and caused by an infection with *Mycoplasma mycoides* subsp.*mycoids* small colony biotype (*MmmSC*). *MmmSC* are resistant to many antibiotics. In regards to this, there is need to develop other antimicrobials from plant origin because of their availability and affordability. It is well established that natural products have been a source of leads for the development of many of the most effective drugs currently available for the treatment of a variety of human diseases. **Experience of participants during the interview**

Participants will interview on their traditional medicines used to control respiratory diseases affecting their cattle and goats; their methods of preparations and the various route of administrations used.

No specific testing will be carried out

No therapy will be given to the participants

The selection of participants will be done randomly

The interviews process to the participants will be done only once

**Potential harm, Injuries, discomforts or inconvenience, risks**

No risk or potential harm will be anticipated as farmers will be interviewed from the homes.

**Potential benefits**

The participants will not benefit directly from participation in the study as the process of drug discovery is a very long process. The 3 years of my PhD might only leads to the discovery of leads compounds which can be considered as the starting point. So I am in a position of making any promise to the participants.

The results of the study could benefit the community after many years

**Confidentiality:** Identities of the participants will not be revealed

**Reimbursement:** N/A

**Participation**

Participants will take part on a voluntary basis and not be paid. There will be no pressure on them to answer the questions and they can refuse to answer any questions they do not feel comfortable with.

**Contact person**

Francisca Kama-Kama (Principal investigator)

E-mail: [franciscayeye@yahoo.fr](mailto:franciscayeye@yahoo.fr) or [francisakamakama@gmail.com](mailto:francisakamakama@gmail.com)

Tel: +254735164218

**Sample consent form**

The study you are about to participate in is on traditional medicine that you use to treat your livestock infected with respiratory (lung) diseases. Should you agree to participate in the study, you will be asked to give the name of the medicinal plants (either alone or combined with others), the specific parts of the plant(s) that you use, the preparation procedure, the route of administration and the dosage. You are free to withdraw or refuse to answer any questions at any time without any consequences and your identity will not be revealed.

Should you agree to participate in the study, please sign your name below, indicating that you have read and understood the nature of the study, your responsibilities as the study participant, the inconveniences associated with voluntary participation in the study and that all your questions and concerns concerning the study have been answered satisfactorily.

You will receive a copy of this consent form to take away with you

--------------------------------------------------- ------------------------------------------------------

Signature of the study participant and date Thumbprint of the study participant and date

---------------------------------------------------

Signature of person obtaining consent and date

-----------------------------------------------------

Signature of witness and date
