# Supplementary material for: Selected ethno-medicinal plants from Kenya with in vitro activity against major African livestock pathogens belonging to the “Mycoplasma mycoides cluster”
Source: J Ethnopharmacol. 2016 Nov 4;192:524–34. doi: 10.1016/j.jep.2016.09.034 (PMC5081062; doi:10.1016/j.jep.2016.09.034)
Supplement: Supplementary file 3 — Supplementary material. Other human conditions treated by the twenty selected medicinal plants. [file mmc3.docx]

**Other human conditions treated by the Selected Medicinal Plants**

| Botanical name/Family/voucher number | Plant part used | Other medicinal use | Mode of preparation | Pharmacological Activity | Major classes of compounds | References |
| --- | --- | --- | --- | --- | --- | --- |
| *Acacia.xanthophloea/*  *Mimosaceae/Fmk2012/1* | Stem bark | Coughs and sore throats | Bark rolled into small ball and chewed to treat coughs and sore throats in humans | Antibacterial agents, cough suppressant | Tannins, saponins and sterols | Wina *et al*., 2010; Rehman *et* *al*., 2011; Solomon-Wisdom & Shittu, 2010; Gakuubi & Wanzala, 2012 |
| *Albizia. coriaria/ Mimosaceae/Fmk2012/2* | Stem bark | Venereal diseases, pneumonia, malaria, menorrhagia, post-partum haemorrhage and sore eyes | Bark infusion for pneumonia and malaria, root decoction for gonorrhea, decoction of bark for menorrhagia and postpartum haemorrhage, dried powdered root is boiled in water and the steam is used to treat sore eyes. | Anticancer, antiplasmodial | Saponins, alkaloids and flavonoids | Johns *et al*.; 1990; Ochwang’i *et al*., 2014; Singab *et al*., 2015; Bunalema *et al*., 2014, Melek *et al*., 2011 |
| *Waburgia. ugandensis/ Canellaceae/Fmk2012/3* | Stem bark, Roots and leaves | Stomachache, toothache, fever, colds, general muscular pains, internal wounds, malaria, appetizer, bacterial infections, coughs and related chest problems, diarrehoea | Infusion of the stem bark for pains, malaria, internal wounds bacterial infections, coughs and related chest problems, decoction of the roots used to treat diarrhea, decoction of the bark combined with leaves used for the treatment of malaria | Antimicrobial, antiplasmodial, painkiller, | Glycosides, saponins, tannins, terpenes, terpenoids and flavonoids | Maobe *et al*., 2013; Wube *et* *al*., 2005; Xu *et al*., 2009 |
| *Olea europaea/ Oleaceae/Fmk2012/4* | Stem and Root barks | Malaria, pneumonia, tapeworm, itchy rashes | Stem bark infusion for the treatment of pneumonia and malaria, an infusion of the bark is taken as remedy for tapeworm, a bark decoction is added to the bark to alleviate itchy rashes | Antioxidant, antimicrobial, antiplasmodial, antihistamine | Phenolic compounds, | Pereira *et al.*, 2007; Wichers *et al.*, 2000; Visioli & Galli, 2002; Meirinhos *et al*., 2005 |
| *Ekebergia capensis/*  *Meliaceae/Fmk2012/5* | Stem bark | Remedy for intestinal warms, dysentery, heartburn, an emetic, chronic coughs, headache, gastritis, skin rashes, fungal infections, acne and abscesses | Leaves decoction for warms, bark decoction for dysentery, heartburn and emetic, roots for coughs and dysentery, bark and roots infusion for headache and gastritis, Poultices powdered bark or bark infusion is applied externally to treat skin rashes, fungal infection acne, boils and abscesses | Painkiller, Antimicrobial, antioxidant and cough suppressant | Limonoids, Terpenoids,  tannins, saponins,  alkaloids and  phenolic compounds | Mulholland and Iourine, 1998; Sewram *et al.*, 2000; Tachibana et al., 1996; Irungu *et al.,* 2014; Amoo *et al*.,2012; Ndukui *et al*., 2012 |
| *carissa.spinarum/Apocynaceae/Fmk 2012/6* | Stem bark, roots and fruits | Painkiller, malaria, ingestion and abdominal pains during pregnancy, | A decoction of roots as painkiller and malaria, taken warm and in small quantities is helpful for ingestion and abdominal pains during pregnancy, fruits for the treatment of dysentery | Painkiller, antiplasmodial agents, antioxidants, antibacterial agents | Tannins, saponins, glycosides, flavonoids anthraquinones, and terpenoids | Maobe *et al*., 2013; Rao *et al*., 2005; Moshi *et al*., 2012 ; Hegde and Joshi, 2010; Gakuubi & Wanzala, 2012 |
| *Tithonia diversifolia/ Asteraceae/Fmk2012/7* | Stem and leaves | Diabetes mellitus, stomach pains, ingestion, sore throats, and liver related problems | An infusion of stem and leaves is used for the treatment of diabetes, stomachache, ingestion sore throats and liver complications | Painkiller, antidiabetes, antimicrobial, hepatoprotective agents | Tannins, saponins, terpenoids | Kuo & Lin, 1999; Ogundare., 2007; Sánchez-Mendoza *et* *al*., 2011 |
| *Euclea divinorum/Ebenaceae/Fmk2012/8* | Stem bark | Purgative, headaches and toothaches, appetizer | Soup made from the bark and roots as purgative, infusion of powdered roots for headaches and toothaches, dried powdered bark infusion as appetizer | Painkiller, appetizer, antioxidants | Tannins, terpenes, naphtoquinones, terpenoids | Hattas *et al.*, 2011 ; Joubert *et al*., 2006 |
| *Piliostigma thonningii/ Fabaceae/Fmk2012/9* | Stem bark, Roots, Twigs and Leaves | Dysentery, fever, respiratory ailments, snake bites, hookworm, skin infections, malaria thirst reliever | Roots and twigs are used as remedy for dysentery, fever, respiratory ailments and snakebites, leaves are edible and chewed by Maasai to relieve thirst | Analgesic agents, painkiller, antidote, antimicrobial | Saponins, flavonoids, phenolics, glycosides, anthraquinones | Jimoh and Oladiji, 2005; Bako *et al*., 2005; Madara *et al*., 2010 |
| *Rhus Vulgaris/ Anacardiaceae/Fmk2012/10* | Roots and fruits | Pains, dental diseases | Roots and fruits decoction used as remedy for diarrhea, stick are used for dental diseases | Antidiarrheal, antibacterial agents | Flavonoids, tannins, saponins, phenolic compounds | Odongo *et al*.,2011; Okullo *et al*., 2014; Fage and Abdullrahman, 2013 |
| *Ziziphus abyssinica/ Rhamnaceae/Fmk2012/11* | Roots, Aerial part | Asthma, pneumonia | Roots decoction taken as remedy for asthma, dried root powdered is rubbed into incisions on the chest to treat pneumonia | Antimicrobial agents, antiasthma, antioxidants | Alkaloids, saponins, flavonoids, tannins, sterols and steroids | Nyaberi *et al*., 2010; Wagate et al., 2008 |
| *Gutenbergia cordifolia/ Asteraceae/Fmk2012/12* | Leaves | Stomachache, malaria | Leaves are pounded and given to children to treat stomachache, ND | Painkiller, antiplasmodial | Terpenes, terpenoids, | Chinsembu, 2015 |
| *Lantana trifolia/Verbenaceae/Fmk2012/13* | Aerial part and/or the whole plants | Remedies for hepatic diseases, glandular disorders, rheumatism, colds, coughs, sore throats, Flu, Malaria, bacterial and fungal infections | Leaves juice or leaves decoction is used as a remedy for hepatic diseases, leaves are pounded, mixed with hot water and applied to sore eyes and treatment of glandular disorders, roots are mixed with hot water and extracts taken as remedy for rheumatism, leaves decoction is used for flu, colds, cough and sore throats  ND | Painkiller, anthelmintic, antiplasmodial, antimalarial, antiviral, antimicrobial, antioxidant, anti-inflammatory, analgesic | Terpenes, terpenoids, steroids, phenolic compounds, flavonoids, glycosides, furanonaphthoquinones, | Johns *et al*., 1990; Sousa and Costa, 2012; Julião *et al.*, 2010; Nalubega et al., 2011; Kisangau *et al*., 2007 |
| *Solanum aculeastrum/ Solanaceae/Fmk2012/14* | Fruits, roots | Contagious bovine pleuropneumonia; pains, gonorrhea | Roots decoction is used to treat gonorrhoea, fruit pulp is applied to warts and bleeding wounds | Antimicrobial, antioxidants, anticancer | Alkaloids, steroidal alkaloids glycosides, | Wanyonyi et al., 2003; Koduru *et al*., 2007 |
| *Garcinia buchananii/ Clusiaceae/Fmk 2012/15* | Stem bark, roots | Chest pain, asthma, diarrhea, abdominal discomfort | Concoction of stem bark and roots is used for the treatment of asthma, chest pain, decoction of stem bark is used to treat diarrhea and abdominal discomfort | Antidiarrheal, anti-motility, antiasthma, analgesic, antiviral, anticancer | Flavonoids, sugars, glycosides, alkaloids, tannins, phenols | Boakye *et al*., 2012; Magadula and Suleimani, 2010; Magadula and Tewtrakul, 2013 |
| *Tylosema fassoglensis/Fabaceae/Fmk2012/16* | Roots | Constipation, stomachache and other gastrointestinal complications, anaemia, fever, jaundice, pneumonia and hypertension | Decoction of roots is used for constipation, stomachache and other gastrointestinal related complications  ND | Antidiarrheal, analgesic, Antimicrobial | Tannins, phenolic compounds, Flavonoids | Adongo *et al*., 2012; Chingwaru *et al*., 2010; Chingwaru *et al*., 2015 |
| *Momordica foestida/ Curcubitaceae/Fmk2012/17* | Aerial parts | Skin rashes, cough, fever, stomachache, intestinal worms, malaria | Young leaves are pounded and apply onto the skin, young leaves are eaten to relieve stomachache and intestinal warms, concoction of the aerial part is used as remedy for fever and cough, ND | Antimalarial, antidiabetic agents, analgesic, cough reliever, antioxidant | Terpenoids, alkaloids, glycosides | Froelich *et al*., 2007; Ntie-Kang *et al*., 2014; Omotayo and Borokini, 2012; Acquaviva *et al*., 2013 Odhiambo *et al*., 2011 |
| *Fuerstia Africana/Lamiaceae/Fmk2012/18* | Aerial part | Stomachache, Urinary infections, ulcers and snakebites | Maceration, concoction, infusion of the leaves is used as remedy for stomachache urinary infections, ulcers and snakebites | Anticatarrhal effect, nerve stimulant, analgesic, antimicrobial, | Tannins, saponins, alkaloids, flavonoids, glycosides, terpenoids, sterols | Okach *et al*., 2013; Muthaura *et al*., 2007; Matu *et al*., 2012 |
| *Toddalia asiática/Rutaceae/Fmk2012/19* | Stem bark, roots and leaves | Malaria, fever, coughs, sore throats, chest pains, food poisoning, rheumatism, Pneumonia and asthma | Root with stem bark concoction is used for malaria and fever, chewing roots helps relieve sore throats, roots bark decoction is used as remedy for chest pains as well as inhaling leaves concoction, roots bark decoction is used for food poisoning, roots, stem and leaves concoction is used for rheumatism and general pains, roots bark concoction is used as treatment for pneumonia and asthma | Antimalarial, analgesic, antimicrobial, antipyretic | Alkaloids, saponins, tannins, steroids, clavonoids, coumarins | Ntie-Kang *et al*., 2014; Duraipandiyan *et al*., 2006 Maobe et al., 2013; Wang, Xu, and Liu, 2009 |
| *Solanum incanum/ Solanacea/Fmk2012/20* | Fruits, roots and leaves | Remedy for warts and bleeding wounds, Snakebite, skin diseases, earache, abdominal pains, fever, stomachache, ingestion, toothache | Fruit pulp used for warts, bleeding wounds and toothache, young leaves chewed and rubbed into the area of snakebite, infusion of leaves administered as remedy for earache, juice from the fruits is applied onto the skin for the treatment of skin diseases, decoction of the roots is taken for abdominal pains, fever, stomachache and ingestion, roots are used to treat toothache | Analgesic, antipyretic, antimicrobial, Antimicrobial, anticancer, | Steroids, glycosides, Alkaloids, flavonoids, saponins | Lin, 2000; Al-Fatimi *et al*., 2007 |

ND: not disclosed (mode of preparation not disclosed)

**Reference**

Acquaviva, R., Di Giacomo, C., Vanella, L., Santangelo, R., Sorrenti, V., Barbagallo,I., … Iauk, L. (2013). Antioxidant activity of extracts of Momordica Foetida Schumach. et Thonn. *Molecules*, *18*, 3241–3249.

Adongo, J. O., Omolo, J. O., Njue, A. W., & Matofari, J. W. (2012). Antimicrobial activity of the root extracts of Tylosema fassoglensis Schweinf . Torre & Hillc ( Caesalpiniaceae ). *Science Journal of Microbiology*, (1), 1–3.

Al-Fatimi, M., Wurster, M., Schröder, G., & Lindequist, U. (2007). Antioxidant, antimicrobial and cytotoxic activities of selected medicinal plants from Yemen. *Journal of Ethnopharmacology*, *111*(3), 657–666.

Amoo, S. O., Aremu, A. O., Moyo, M., & Van Staden, J. (2012). Antioxidant and acetylcholinesterase-inhibitory properties of long-term stored medicinal plants. *BMC Complementary and Alternative Medicine*, *12*(1), 87.

Bako S.P, Bakur M.J, J. I. and B. E. I. (2005). Ethonomedicinal and Phytochemical profile of some Savanna Species in Nigeria. *International Journal of Botany*, *1(2)*, 147–150.

Boakye, P. a, Stenkamp-Strahm, C., Bhattarai, Y., Heckman, M. D., Brierley, S. M., Pasilis, S. P., & Balemba, O. B. (2012). 5-HT(3) and 5-HT(4) receptors contribute to the anti-motility effects of Garcinia buchananii bark extract in the guinea-pig distal colon. *Neurogastroenterology and Motility : The Official Journal of the European Gastrointestinal Motility Society*, *24*(1), e27–40.

Bunalema, L., Obakiro, S., Tabuti, J. R. S., & Waako, P. (2014). Knowledge on plants used traditionally in the treatment of tuberculosis in Uganda. *Journal of Ethnopharmacology*, *151*(2), 999–1004.

Chingwaru (2011). Antibacterial and anticandidal activity of *Tylosema esculentum* (marama) extracts. *South African Journal Sciences*, 107 (3/4)

Chingwaru, W., Vidmar, J., Kapewangolo, P. T., Mazimba, O., & Jackson, J. (2015). Therapeutic and Prophylactic Potential of Morama ( Tylosema esculentum ): A Review. *Phytotherapy Research*,

Chinsembu, K. C. (2015). Plants as antimalarial agents in Sub-Saharan Africa. *Acta Tropica*, *152*, 32–48.

Duraipandiyan, V., Ayyanar, M., & Ignacimuthu, S. (2006). Antimicrobial activity of some ethnomedicinal plants used by Paliyar tribe from Tamil Nadu, India. *BMC Complementary and Alternative Medicine*, *6*, 35.

Fage, Z., & Abdullrahman, A. (2013). SCREENING IN VITRO AND IN VIVO THE ANTIBACTERIAL ACTIVITY OF RHUS CORIARIA EXTRACT AGAINST S . AUREUS. *IJRRAS*, *15*(1), 390–397.

Froelich, S., Onegi, B., Kakooko, A., Stems, R., Schubert, C., & Jenett-Siems, K. (2007). Plants traditionally used against malaria: Phytochemical and pharmacological investigation of Momordica foetida. *Brazilian Journal of Pharmacognosy*, *17*(1), 1–7.

Gakuubi, M. M., & Wanzala, W. (2012). A survey of plants and plant products traditionally used in livestock health management in Buuri district, Meru County, Kenya. *Journal of Ethnobiology and Ethnomedicine*, *8*(1), 39.

Hattas, D., Hjältén, J., Julkunen-tiitto, R., & Peter, F. (2011). Author ’ s personal copy Differential phenolic profiles in six African savanna woody species in relation to antiherbivore defense. *Phytochemistry*, *72*, 1796–1803.

Hegde, K., & Joshi, A. B. (2010). Hepatoprotective and antioxidant effect of Carissa spinarum root extract against CCl 4 and paracetamol-induced hepatic damage in rats. *Bangladesh Journal of Pharmacology*, *5*, 73–76.

Irungu, B., Orwa, J., Gruhonjic, A., Fitzpatrick, P., Landberg, G., Kimani, F., … Yenesew, A. (2014). Constituents of the Roots and Leaves of Ekebergia capensis and Their Potential Antiplasmodial and Cytotoxic Activities. *Molecules*, *19*(9), 14235–14246.

Jimoh F O and Oladiji A.T. (2005). Preliminary Studies on Piliostigma thonningii seeds : Proximate analysis , mineral composition and phytochemical screening. *Africa Journal of Biotechnology*, *4*(December), 1439–1442.

Johns, T., Kokward, J. O., & Kimanani, E. K. (1990). Herbal remedes of the Luo of Siaya district Kenya: Establishing quantittive criteria for consensus. *Economic Botany*, *44*(3), 369–381.

Joubert, a., Kooy, F., Meyer, J. J. M., & Lall, N. (2006). HPLC in the Comparative Study of the Content of Naphthoquinones (Quinonoid Constituents) in Euclea Species of South Africa. *Chromatographia*, *64*(7-8), 399–403.

Julião, L. de S., Leitão, S. G., Lotti, C., Picinelli, A. L., Rastrelli, L., Fernandes, P. D., … Leitão, G. G. (2010). Flavones and phenylpropanoids from a sedative extract of Lantana trifolia L. *Phytochemistry*, *71*(2-3), 294–300.

Kisangau, D. P., Hosea, K. M., Joseph, C. C., & Lyaruu, H. V. M. (2007). In vitro antimicrobial assay of plants used in traditional medicine in Bukoba rural district, Tanzania. *African Journal of Traditional, Complementary and Alternative Medicines*, *4*(4), 510–523.

Koduru Srinivas, Jimoh F.O, G. D. . and A. A. . (2007). Antioxidant Activity of two Steroid Alkaloids extracted from Solanum culeastrum. *Journal of Pharmacology and Toxicology*.

Kuo, Y. H., & Lin, B. Y. (1999). A new dinorxanthane and chromone from the root of Tithonia diversifolia. *Chemical & Pharmaceutical Bulletin*, *47*(3), 428–429.

Lin, Y. (2000). Nonsteroidal Con stit u ents from Solanum incanum L . *Journal of the Chemical Society Chinese*, *47*, 247–251.

Madara, A. A., Ajayi, J. A., Salawu, O. A., & Tijani, A. Y. (2010). Anti-malarial activity of ethanolic leaf extract of Piliostigma thonningii Schum . ( Caesalpiniacea ) in mice infected with Plasmodium berghei berghei. *Afrcian Journal of Biotechnology*, *9*(23), 3475–3480.

Magadula, J. J., & Tewtrakul, S. (2013). Anti-HIV-1 protease activities of crude extracts of some Garcinia species growing in Tanzania. *African Journal of Biotechnology*, *9*(12), 1848–1852.

Magadula, J., & Suleimani, O. (2010). Cytotoxic and anti ‐ HIV activities of some Tanzanian Garcinia species. *Health (San Francisco)*, *12*(2), 1–7.

Maobe, M. A. G., Gitu, L., Gatebe, E., Rotich, H., Karanja, P. N., Votha, D. M., … Muingai, C. (2013). Antimicrobial activities of eight selected medicinal herbs used for the treatment of diabetes, malaria and pneumonia in kisii region,southwest Kenya. *Global Journal of Pharmacology*, *7*(1), 25–33.

Matu, E. N., Kirira, P. G., Kigondu, E. V. M., Moindi, E. M., & Amugune, B. A. (2012). Antimicrobial activity of organic total extracts of three Kenyan medicinal plants. *African Journal of Pharmacology and Therapeutics*, *1*(1), 14–18.

Meirinhos, J., Silva, B. M., Valentão, P., Seabra, R. M., Pereira, J. a, Dias, A., … Ferreres, F. (2005). Analysis and quantification of flavonoidic compounds from Portuguese olive (Olea europaea L.) leaf cultivars. *Natural Product Research*, *19*(2), 189–195.

Melek, F. R., Ghaly, N. S., & Nabil, M. (2011). Flavonoids from Albizia procera. *Egytian Journal of Pure and Applied Sciences*, 79–82.

Moshi, M. J., Otieno, D. F., & Weisheit, A. (2012). Ethnomedicine of the Kagera Region, north western Tanzania. Part : plants used in traditional medicine in Katoro village, Bukoba District. *Journal of Ethnobiology and Ethnomedicine*, *8*(1), 14. Mulholland, D. A., & Iourine, S. E. (1998). LIMONOIDS FROM EKEBERGIA CAPENSIS. *Phytochemistry*, *47*(7), 1357–1361.

Muthaura, C. N., Keriko, J. M., Mutai, C., Yenesew, A., Gathirwa, J. W., Irungu, B. N., … Derese, S. (2007). Antiplasmodial potential of traditional antimalarial phytotherapy remedies used by the Kwale community of the Kenyan Coast. *Journal of Ethnopharmacology*, *170*(3), 148–57.

Nalubega Rebecca, Kabasa David John, O. D. and K. J. (2011). Antibacterial and Phytochemical Screening of Eleven Plants Used as Poultry Ethnomedicines in Southern Uganda. *Agricultural Journal*, *6(6)*(1816-9155), 303–309.

Ndukui, J. G., Muwonge, H., & Sembajwe, L. (2012). Aphrodisiac potential and phytochemical profile of Ekebergia capensis (Cape ash) in male albino rats. *Spatula DD - Peer Reviewed Journal on Complementary Medicine and Drug Discovery*, *2*(4), 237–243.

Ntie-Kang, F., Onguéné, P. A., Lifongo, L. L., Ndom, J. C., Sippl, W., & Mbaze, L. M. (2014). The potential of anti-malarial compounds derived from African medicinal plants, part II: a pharmacological evaluation of non-alkaloids and non-terpenoids. *Malaria Journal*, *13*(1), 81.

Nyaberi, M. O., Onyango, C. A., Mathooko, F. M., Maina, J. M., Makobe, M., & Mwaura, F. (2010). Evaluation of phytochemical, antioxidant and antibacterial activity of edible fruit extracts of Ziziphus abyssinica A. Rich. *Journal of Animal & Plant Sciences*, *6*(2), 623–629.

Ochwang’i, D. O., Kimwele, C. N., Oduma, J. A., Gathumbi, P. K., Mbaria, J. M., & Kiama, S. G. (2014). Medicinal plants used in treatment and management of cancer in Kakamega County, Kenya. *Journal of Ethnopharmacology*, *151*(3), 1040–1055. Odhiambo, J. A., Lukhoba, C. W., & Dossaji, S. F. (2011). Evaluation of herbs as potential drugs/medicines. *African Journal of Traditional, Complementary, and Alternative Medicines : AJTCAM / African Networks on Ethnomedicines*, *8*(5 Suppl), 144–51.

Odongo, C. O., Musisi, N. L., Waako, P., & Obua, C. (2011). Chewing-stick practices using plants with anti-streptococcal activity in a Ugandan rural community. *Frontiers in Pharmacology*, *MAR*(March), 1–5.

Ogundare., 2007. (2007). Antimicrobial Effect of Tithonia diversifolia and Jatropha gossypifolia leaf extracts. *Trends in Applied Sciences Research*, *2*(2), 145–150.

Okach, D. O., Nyunja, A. R. O., & Opande, G. (2013). Phytochemical screening of some wild plants from Lamiaceae and their role in traditional medicine in Uriri District - Kenya, *1*(5), 135–143.

Okullo, J. B. L., Omujal, F., Bigirimana, C., Isubikalu, P., Malinga, M., Bizuru, E., & Namutebi, A. (2014). Journal of Medicinal Plants Studies Ethno-Medicinal Uses of Selected Indigenous Fruit Trees from the Lake Victoria Basin Districts in Uganda, *2*(1), 78–88.

Omotayo, F. O., & Borokini, T. I. (2012). Comparative phytochemical and ethnomedicinal survey of selected medicinal plants in Nigeria. *Scientific Research and Essays*, *7*(9), 989–999.

Pereira, A. P., Ferreira, I. C. F. R., Marcelino, F., Valentão, P., Andrade, P. B., Seabra, R., … Pereira, J. A. (2007). Phenolic compounds and antimicrobial activity of olive (Olea europaea L. Cv. Cobrançosa) leaves. *Molecules (Basel, Switzerland)*, *12*(5), 1153–1162.

Rao, R. J., Kumar, U. S., Reddy, S. V., Tiwari, A. K., & Rao, J. M. (2005). Antioxidants and a new germacrane sesquiteroene from Carissa spinarum. *Natural Product Research*, *19*(8), 763–769.

Rehman, S., Ashfaq, U. A., Riaz, S., Javed, T., & Riazuddin, S. (2011). Antiviral activity of Acacia nilotica against Hepatitis C Virus in liver infected cells, 1–6. Sánchez-Mendoza, M. E., Reyes-Ramírez, A., Cruz Antonio, L., Martínez Jiménez, L., Rodríguez-Silverio, J., & Arrieta, J. (2011). Bioassay-guided isolation of an anti-ulcer compound, tagitinin C, from Tithonia diversifolia: role of nitric oxide, prostaglandins and sulfhydryls. *Molecules (Basel, Switzerland)*, *16*(1), 665–74.

Sewram, V., Raynor, M. W., Mulholland, D. A., & Raidoo, D. M. (2000). The uterotonic activity of compounds isolated from the supercritical fluid extract of Ekebergia capensis. *Journal of Pharmaceutical and Biomedical*, *24*, 133–145.

Singab AN, Bahgat D, A.-S. E. and E. O. (2015). Saponins from Genus Albizia: Phytochemical and Biological Review. *Medicinal and Aromatic Plants*, 1–7. Solomon-Wisdom, G. O., & Shittu, G. A. (2010). In vitro antimicrobial and phytochemical activities of Acacia nilotica leaf extract. *Journal of Medicinal Plants Research*, *4*(12), 1232–1234.

Sousa, E. O., & Costa, J. G. M. (2012). Genus Lantana: Chemical aspects and biological activities. *Brazilian Journal of Pharmacognosy*, *22*(5), 1155–1180.

Tachibana, Y., Kato, A., Mathenge, S. G., Nganga, J. N., & Juma, F. D. (1996). Acyclic triterpenoids from ekebergia capensis. *Phytochemistry*, *42*(3), 803–807.

Visioli, F., & Galli, C. (2002). Biological properties of olive oil phytochemicals. *Critical Reviews in Food Science and Nutrition*, *42*(April 2015), 209–221.

Wagate Cyrus;, Daniel, G. W., Nanyingi, M. O., Njonge, F. K., & Mbaria, J. M. (2008). Antibacterial and cytotoxic activity of Kenyan medicinal plants. *Mem Inst Oswaldo Cruz, Rio de Janeiro*, *103*(7), 650–652.

Wang, F., Xu, Y., & Liu, J.-K. (2009). New geranyloxycoumarins from Toddalia asiatica. *Journal of Asian Natural Products Research*, *11*, 752–756.

Wanyonyi, A. W., Tarus, P. K., & Chhabra, S. C. (2003). A novel glycosidic steroidal alkaloid from Solanum aculeastrum. *Bulletin of the Chemical Society of Ethiopia*, *17*(1), 61–66.

Wichers, H. J., Soler-rivas, C., & Espı, J. C. (2000). Review Oleuropein and related compounds. *Journal of the Science of Food and Agriculture*, *80*(November 1999), 1013–1023.

Wina, E., I W R, S., & Tangendjaja, B. (2010). Biological Activity of Tannins from Acacia mangium Bark Extracted by Diff erent Solvents. *Media Peternakan*, *33*(2), 103–107.

Wube, A. A., Bucar, F., Gibbons, S., & Asres, K. (2005). Sesquiterpenes from Warburgia ugandensis and their antimycobacterial activity. *Phytochemistry*, *66*(19), 2309–2315.

Xu, M., Litaudon, M., Krief, S., Martin, M.-T., Kasenene, J., Kiremire, B., … Guéritte, F. (2009). Ugandenial A, a new drimane-type sesquiterpenoid from Warburgia ugandensis. *Molecules (Basel, Switzerland)*, *14*(10), 3844–50.
