# Supplementary material for: Selected ethno-medicinal plants from Kenya with in vitro activity against major African livestock pathogens belonging to the “Mycoplasma mycoides cluster”
Source: J Ethnopharmacol. 2016 Nov 4;192:524–34. doi: 10.1016/j.jep.2016.09.034 (PMC5081062; doi:10.1016/j.jep.2016.09.034)
Supplement: Supplementary file 4 — Supplementary material. Results of the total number of the crude extracted from the selected twenty plants. [file mmc4.docx]

Supplementary 4

| Plant name | Plant parts | Numbers of extracts | Total numbers of extract par plant |
| --- | --- | --- | --- |
| *A. xenthophloeae* | Stem bark | 4 | 8 |
|  | Roots | 4 |  |
| *W.ugandensis* | Stem bark | 4 | 8 |
|  | Roots | 4 |  |
| *O. europaea* | Stem bark | 4 | 8 |
|  | Roots | 4 |  |
| *M. foetida* | Whole plant | 4 | 4 |
| *S.incanum* | Stem bark | 4 | 8 |
|  | Leaves | 4 |  |
| *R.vulgaris* | Stem bark | 4 | 8 |
|  | Leaves | 4 |  |
| *A.coriaria* | Stem bark | 4 | 8 |
|  | Leaves | 4 |  |
| *S.aculeastrum* | Stem bark | 4 | 12 |
|  | Berries | 4 |  |
|  | Leaves | 4 |  |
| *C.spinarum* | Stem bark | 4 | 8 |
|  | Leaves | 4 |  |
| *G.cordifolia* | Leaves | 4 | 8 |
|  | Roots | 4 |  |
| *T. diversifolia* | Stem bark | 4 | 8 |
|  | Leaves | 4 |  |
| *T.asiatica* | Stem | 4 | 8 |
|  | Leaves | 4 |  |
| *F. africana* | Whole plant | 4 | 4 |
| *L.trifolia* | Whole plant | 4 | 4 |
| *G. buchananii* | Stem bark | 4 | 8 |
|  | Leaves | 4 |  |
| *T.fassoglensis* | Stem | 4 | 8 |
|  | Leaves | 4 |  |
| *Z. abyssinica* | Stem bark | 4 | 8 |
|  | Leaves | 4 |  |
| *E. capensis* | Stem bark | 4 | 8 |
|  | Leaves | 4 |  |
| *P.thonningii* | Stem bark | 4 | 8 |
|  | Leaves | 4 |  |
| *E.divinorum* | Stem | 4 | 4  4 |
|  | Leaves | 4 |  |
| Total numbers of extracts | - | - | 152 |

Table A. Results showing total numbers of extracts
