# Supplementary material for: Selected ethno-medicinal plants from Kenya with in vitro activity against major African livestock pathogens belonging to the “Mycoplasma mycoides cluster”
Source: J Ethnopharmacol. 2016 Nov 4;192:524–34. doi: 10.1016/j.jep.2016.09.034 (PMC5081062; doi:10.1016/j.jep.2016.09.034)
Supplement: Supplementary file 6 — Supplementary material. Luo translation. [file mmc6.docx]

**Dwaro /manyo yiend/yedhe bungu mathiedho touché oboye mag dhok gi diek**

**Jononro**

**Francisca kama(Japuonjre matiegre e rang’iny mar PhD e kar tiegruok ma JKUAT kendo Ja**

**nonro motelo maduong’)**

**Laktar /ajuoga Joerg Jones (manyawadgi Janonro kendo nyapara e JKUAT)**

**Kar tiegruok: JKUAT/ILRI**

**Dwach nonro**

Thor mar tiegruok e manyo/dwaro yien/yedhe makelo lokruok e del kendo tamo touche mabiro eyor mulo ng’ato kod mikeyo bende.

**Tiegruok mar kit gimoro**

Le man go kido achiel man gi nyii gimoro achiel mar gimiluongo n kido mochan maber man konchiel kendo duto beyo. Gin gik moko ma ok mapile machung mag Jamni duto kaka diek gi tuoche mag oboye mag dhok ,touché malandre modok kor kadhok makelo pek eyweyo gi ranyisi mar pek yweyo, gin touché madongo mag Jamni kendo ohinyo sekta mar pur to moloyo e pinje madongore ei ohala mag gik mowuok kuom Jamni dko chien mar keyo chiemo kod dok chien mar nyuolruok. E gimilosoni,wabiroketo pachwa e tuochemalandre mag pek mar yweyo dhok ma en achiel kuom tuoche madongo malandre mahinyo dhok e Afrika. Tuoche malandre pek mar yweyo ikelo gi tuo mochiw gi gik machal kendgi. Tong kutegi gin chokruok matin mar gik kedo. Gini rateng mag nego bakteria kendo thiedho tuo.

Kaluore gima nitie dwaro mar dongo gik moko man gi chal mopogore gi mamoko mapile kuom chwech yien nikech bedogi kod nyalruok. En gima olos maber manyuolre mag gik mochwe osebedo kar chokruok mar gi, nyis kuom dongruok mar ng’eny mag yien /yedhe mag teko to moloyo kinde masani mayudre kuom thieth mag touché mathoth mahinyo dhano.

**Lony mar Jomabiro chiwo paro e kinde mag mich penj**

Jomabiro chiwo parogo biro penjo kuom yiend nyaluo kata mag bungu mitiyogo e geng’o touché makelo pek eyweyo mahinyo dhogi gi diek;chenro ma gi mag ikruok kod yore mopogre mag rit gik tich motigo.

Onge non mong’ere mibiro timo onge thieth mibichiw ni Jochiw paro yiero mar jochiw paro ibi tim apoya ka gi kacha.

Kaka ibi yiero jochiw paro ibi tim dichiel

**Chwanyruok manyalo betie , hinyruok kata winjo marach, chandruok**

Onge chandruok kata chwanyruok manyalo betie mibigen kaka Jopur ibi penj koa e mie

**Ohala /yuto manyalo betie**

Jochiw paro ok bi yuto e yo moriere kuom chip paro e tiegruok kaka fweny timo gikmaok mag fweny yien/yedhe en timo gik moko eyo mabor. Hika adek mag PhD nyalo bedo mana ranyisi e fweny mar nyisruok gik mopogore manyalo paro kaka chakruok moyiedhi. kuom mano ane rang’iny mar chiwo singo/singruok moro amora ni Jochiw paro. Duoko mar tiegruok nyalo konyo Jodak kanyakla bang higni mang’eny.

**Malingling gikmoko**:chal mar Jochiw paro ok bi nyis

**Dwoko manyonge**: Gi mamoko

**Chiwo mag paro**

Jochiw paro biro betie eyor chiwruok kendo ok bi chulgi. Ok bi betie achune kuomgi e duoko penjo kendo ginyalo dagi duoko penjo moro amora magiwinjo ni ok owinjre kodgi.

Ng’ama inyalo tudruokgo

Francisca Kama-Kama (Janonro maduong)

Mbui: Franciscayeye @yahoo.fr or [Francisakamakama@gmail.com](mailto:Francisakamakama@gmail.com)

Simo:+2547355164218

**Fom mar yie chal gimoro kata winjruok**

Tiegruok michiegni betie e wi yiend bungu mitiyogo e thiedho Jambi man gi tuo mar pek yweyo mag oboye: Kinyalo yie betie e tiegruok ibi penji mondo ichiw nying mar yiend bungu(kendi kata koriwre gi mamoko), bethegi mong’ere mitiyogo, chal ikogi kata losogi yor ritogi to kod rapim yath. In thuolo mar wuok kata tamri duoko penjo moko amoka e sa asaya maonge kum moko kendo,chalni ok binyis.

Kiyie ni inyalo betie e tiegruok, kiyie to ket kata go sei e bwo nyingi nyis ni isesomoo kendo iwinjo tiend chal tiegruok, tijni kaka Jachiw paro mar tiegruok; Winjo marach maluore gi chiwruok maok ochun e tiegruok kendo penjoni duto gi dwach gimoro maluore gi tiegruok oseduok eyo makare.

Ibiro yudo fwanu mar fom winjruogni midhigo.

***Sei mar Jachiwparo etiegruok gi tarik***

***Mhuri mar kogno mathoun mar Jachiwparo etigruok gi tarik***

***Sei mar ng’ama yudo winjruok gi tarik***

***Sei mar Janeno gi tarik***
