# Supplementary material for: Selected ethno-medicinal plants from Kenya with in vitro activity against major African livestock pathogens belonging to the “Mycoplasma mycoides cluster”
Source: J Ethnopharmacol. 2016 Nov 4;192:524–34. doi: 10.1016/j.jep.2016.09.034 (PMC5081062; doi:10.1016/j.jep.2016.09.034)
Supplement: Supplementary file 7 — Supplementary material. Maasai translation. [file mmc7.docx]

**ELUK UNYA**

Embolokinoto emakewan oolajurrok le nkeeya oolkipieu toonkishu wesipata olchani loonkaitubulu ogeluno  tolosho le Kenya ogilunore nkurt naayau ina moyian oolkipeu

**ILAJURROK**

**Fransisca Kama-Kama enkerai  naasita PhD te JKUAT  olkitok lenjurrorre.**

**Dr Joerg Jores tenebo ilaing’urak olajurrok le ILRI.**

**Dr Joseph Nganga le JKUAT laa tenebo lelo ajurrok.**

**ENKIPIRTA ENJURRORE**

Ore enkipirta ena jurrore naa pee eibalieki tipat tialo ilkeek oopaasha anaa ooidim aatabak enkeeya oolkipieu werishata olchani leina moyian.

**EWALATA ENKITENG’ENARE**

Ore ele shani le moyian oolkipieu naa nabo o kulikae keek  naa iletipat keidimu sii imoyiaritin oo nkineji o nkishu, ore sii likae shani oidim aibooi emoyian oloirobi toontokitin naaramati,naa ilkeek looswam tenebo wenturore neretu sii maendeleo olosho eiimu oramatie loonkishu ondaiki tembulunoto, ore anaa enikidolita enkeeya oolkipieu aa ninye emoyian sapuk toonkishu tolkila orok naa keatae enkurto naitasuroo ena moyian. keetae eyieunoto enkibelekenyata tialo nena moyiaritin toondaiki tenkaraki enkidimata wembulunoto oramatie, etujurroki nchere ore imasaa arashu indaiki anaa ilng’anayio neeta ilkeek oramatieki pee eidimie metubulu neitapaash imoyiaritin naijo inooltung’anak.

**EYIOLOUNOTO TELELO OOTABAITIE ENKIKILIKUANISHORE**

Keyiolou ilkeek oiboorieki  imoyiaritin oonkishu o nkineji tiaalo olkuak lenye, enkoitoi o barata nabakishoreki.

Imeibala orreikie tialo ina baare

Imeetae ewalet naishori lelo  aikilikuanak

Mmetii enkoitoi naing’ori pee egeluni  iloopuo aas ina siai

Enkata nabo ake epuoi enkikilikuanare

**ENKURETISHO, ILBAA, BATISHO**

Mmeetae batisho tialo ilaramatak naidimayu pee eitanyamalisho.

**DUPOTO**

Elelek medupoyu lelo oopuo enkiteng’enare tenkaraki neshomo kake keetae enkoitoi naado eyiolounoto elelo keek, eidimayu paa ore enkisuma o ilarin okuni  le PhD naa enkiterunoto e njurrunoto, kedupore iltung’anak ena kiteng’enare tiatua ilarin kumok**.**

**ILOOJING’ ILAIKITENG’ENAK**

Keitayu ate ilelo oopuo ina kiteng’enare melakitae, meeta sii engolon naolie ashu naolikinyieki eidimayu nemeyiolo.

**TOOSHOKI**

**Fransisca Kama-Kama( orkitok lenjurrore)**

**E-mail: [fransisca+eye@yahoo.fr](mailto:fransisca+eye@yahoo.fr) or [fransiscakamakama@gmail.com](mailto:fransiscakamakama@gmail.com)**

**Tel. +254735164218**

**ENKITANYAANYUKOTO**

Keidimayu pee ejur tialo ilkeek lolkuak oidim aatabaiki inkishu tialo imoyiaritin oo nkishu.

Enkining’okino pee elo aas enjurrore neikilikuanu inkarn elelo keek( aa obo arashu eshula) erreten wenkoitoi. Keidimayu pee minyorraa arashu iyany iwol enkikilikuanata  mmetii enkop pookin.

Teniyieu nilo aas ena jurrore ashu kining’okino tenakisuma tisira ena palai enkarna ino, ingero sii itayiolo nimbung’a ena kisuma, wesiai ino tena jurrore, enyorrakino wenkitainoto ekewan tialo ena kiteng’enare.

**Ekinchori empalai nabo te kuna naaku enino**

**……………………………………………………**

**Enitukuny  tenebo ontarikini**

**………………………………………………**

**Enetukuny olikinkilikuanishore ontarikini**

**……………………………………………….**

**Enetukuny olchakeni ontarikini**

**…………………………………………………**
